# Concepts, Challenges, and Successes in Modeling Thermodynamics of Metabolism

**DOI:** 10.3389/fbioe.2014.00053

**Published:** 2014-11-26

**Authors:** William R. Cannon

**Affiliations:** ^1^Computational Biology and Bioinformatics Group, Biological Sciences Division, Pacific Northwest National Laboratory, Richland, WA, USA

**Keywords:** statistical thermodynamics, metabolism, simulations, fluctuation theory, molecular motors, tricarboxylic acid cycle, adaptation, biological

## Abstract

The modeling of the chemical reactions involved in metabolism is a daunting task. Ideally, the modeling of metabolism would use kinetic simulations, but these simulations require knowledge of the thousands of rate constants involved in the reactions. The measurement of rate constants is very labor intensive, and hence rate constants for most enzymatic reactions are not available. Consequently, constraint-based flux modeling has been the method of choice because it does not require the use of the rate constants of the law of mass action. However, this convenience also limits the predictive power of constraint-based approaches in that the law of mass action is used only as a constraint, making it difficult to predict metabolite levels or energy requirements of pathways. An alternative to both of these approaches is to model metabolism using simulations of states rather than simulations of reactions, in which the state is defined as the set of all metabolite counts or concentrations. While kinetic simulations model reactions based on the likelihood of the reaction derived from the law of mass action, states are modeled based on likelihood ratios of mass action. Both approaches provide information on the energy requirements of metabolic reactions and pathways. However, modeling states rather than reactions has the advantage that the parameters needed to model states (chemical potentials) are much easier to determine than the parameters needed to model reactions (rate constants). Herein, we discuss recent results, assumptions, and issues in using simulations of state to model metabolism.

## Introduction

Since the time of Boltzmann, it was recognized that living organisms are thermodynamic entities. Lotka (1922a) paraphrased Boltzmann’s thinking, “that the fundamental object of contention in the life-struggle, in the evolution of the organic world, is available energy.” Lotka went on, “in accord with this observation is the principle that, in the struggle for existence, the advantage must go to those organisms whose energy-capturing devices are most efficient in directing available energy into channels favorable to the preservation of the species.” Lotka (1922b) proposed that natural selection is at its most fundamental level a physical principle. Schrödinger (1945) famously expanded on this concept with *What is Life?*, and used the concept of entropy to describe how order, in the form of high energy compounds in the environment, drives organization within organisms. Organisms dissipate that energy into lower forms. The concept of life as a non-equilibrium process has resonated with others as well, including Prigogine who described living organisms as dissipative structures that self-organize in response to large non-equilibrium driving forces (Prigogine, 1978). Abiotic examples of dissipative structures include tornadoes, hurricanes, and convection cells. The non-equilibrium driving forces “pay” for the self-organization that allows the resulting structures to dissipate energy rapidly. In biological systems, energy comes into the system in the form of sunlight or high energy compounds, typically highly reduced carbon compounds, and this energy is dissipated into the environment according to the second law of thermodynamics. In biological systems, some of the energy is harvested to pay for the creation of additional dissipative structures (growth and reproduction), or to create large amounts of stored energy in the form of lower energy byproducts.

The ecologist H. T. Odum was certainly convinced of the role of statistical thermodynamics in systems ecology. Writing in the American Scientist (Odum and Pinkerton, 1955), Odum sought to understand the diverse scale of rates of natural processes, and proposed that each biological system works at an efficiency that allows the maximum efficiency and power, similar to Lotka’s concept that the advantage goes to organisms whose metabolism is most efficient at channeling energy for the purpose of reproduction. Odum took natural selection to mean “the persistence of those forms, which can command the greatest useful energy per unit time.”

Morowitz also proposed that the far from equilibrium natural environment was responsible for self-organization of biological systems. As a consequence, Morowitz proposed that life was not only a consequence of energy flow in natural systems, but also that it is highly probable. From this perspective, natural selection is a random process, and in the words of Dewar (2005), species “are selected because they are characteristic of each of the overwhelming majority of ways in which energy and matter could flow under the constraints imposed by local energy and mass conservation”. Such concepts have led to the metabolism first hypothesis of the emergence of life on earth (Smith and Morowitz, 2004).

While an excellent collection of discussions of entropy production and self-organization of natural systems has been presented in the literature (Kleidon et al., 2010), for the most part the recognition by physical scientists of the role of thermodynamics as a causal factor in the operation of biological systems stands in stark contrast to the lack of discussion of thermodynamics in the experimental life sciences literature. A major reason for this may be because of the abstract nature of statistical thermodynamics and the lack of tools to model and evaluate the thermodynamic aspects of living systems. After all, since its conceptualization developments in thermodynamics have had mostly to do with equilibrium processes, and biological systems are highly non-equilibrium.

However, in the last 20 years, statistical thermodynamics and fluctuation theorems have allowed for significant progress in understanding non-equilibrium systems. Fluctuation theorems are starting to be used to model biological systems, allowing us to begin to understand how cellular machinery operates. These theorems tell us that there is an important difference between thermodynamic models of macroscopic process and the statistical thermodynamic models of the microscopic processes such as those that make up cells. The second law of thermodynamics describes macroscopic processes and states that the entropy of a spontaneous process never decreases. The second law is silent, however, about the microscopic events that make up the macroscopic process. These microscopic events may be, for instance, sets of coupled reactions that lead to some observable change of state – a different phenotype in the parlance of biology. These microscopic events involve enzyme complexes and coupled reaction pathways in cells, which are not just scaled down versions of beaker-sized laboratory systems. Components of small systems can in fact run in reverse at times. A number of excellent reviews of fluctuation theorems exist in the literature (Harris and Schutz, 2007; Sevick et al., 2008; Seifert, 2012) and we will only give an in-a-nutshell perspective here.

In this report, we will focus on issues and challenges in thermodynamically modeling biological systems of coupled reactions, such as those that occur in metabolism. We will first discuss probability density functions based on Boltzmann probabilities and the relationship to free energy. Closely related to free energy is the concept of entropy. We will discuss different formulations of entropy and their meanings in order to provide a clear overview of entropy production. Finally, fluctuation theorems will be briefly discussed using this conceptual framework. While fluctuation theorems have not yet been used to extensively simulate metabolism, they have great promise, and have been used to examine single molecule dynamics and the dynamics of coupled biochemical reactions on multiple scales. Finally, the application of statistical thermodynamics to model biological reactions that are far from equilibrium is discussed.

## Theoretical Background

Understanding the foundational concepts of modeling thermodynamics is essential for understanding the challenges that the field faces. The mathematical concepts presented in the literature are often too abstract to be readily accessible to those outside the specialty field of statistical thermodynamics. A case in point is that it may seem like the literature contains a zoo of seemingly unrelated statistics all going by the name of entropy. Understanding which entropy is being used is critical for understanding and applying thermodynamic modeling and fluctuation theorems, as will become evident below.

However, a tremendous amount of physical insight into fluctuation theorems and thermodynamic modeling can be obtained if one understands the multinomial distribution function, which is simply a generalization of the common binomial distribution function when more than two outcomes are possible. With regard to reaction kinetics, more than two outcomes are possible when we have more than two interconverting species present. The mathematical form of a multinomial distribution is,

$$\mathrm{Pr}\;\left(n_{1},\;\ldots ,\;n_{m}|N_{\mathrm{total}},\;\theta_{1},\;\ldots ,\;\theta_{m}\right)=N_{\mathrm{total}}!\prod_{\mathrm{objectsj}}^{m}\frac{1}{n_{j}!}\theta_{\text{j}}^{n_{\text{j}}}.$$

The multinomial probability density above is the probability that *n_j_* objects of type *j* will be present when there are *N*_total_ = Σ*n_j_* objects present. In the equation above, θ*_j_* is the probability of object *j* independent of the other objects. According to frequentist statistics, this probability is simply the long term proportion of the number of object *j*’s that are present, θ*_j_* = *n_j_*/*N*_total_. The probability density is not simply $\mathrm{Pr}=\Pi_{j}\theta_{\text{j}}^{n_{\text{j}}}$ because each individual object of type *j* is indistinguishable from all the other objects of type *j*. Thus, the probability density has to be corrected for the number of permutations and combinations of each object type, which is accounted for by the factorial terms in the multinomial distribution function.

Now consider a system consisting of three chemical species *A, B*, and *C* in aqueous solution in a container of fixed volume. Each of the three species can interconvert to one of the other two species, but the total number of particles is fixed such that *n*_A_ + *n*_B_ + *n*_C_ = *N*_total_. The Boltzmann probability θ*_i_* of species *i* is related to the Helmholtz free energy of solvation $\Delta {𝒜}_{i}^{0}$ by,

(1) $$\theta_{i}=\frac{e^{-\Delta {𝒜}_{i}^{0}∕k_{B}T}}{\sum_{\mathrm{species j}}^{m}e^{-\Delta {𝒜}_{j}^{0}∕k_{B}T}}.$$

where *k_B_* is Boltzmann’s constant and *T* is the temperature. For simplicity, we will disregard the internal degrees of freedom for each species. In this case, the numerator $e^{-\Delta {𝒜}_{i}^{0}∕kT}$ is referred to as the molecular partition function, *q_i_*. The denominator is simply a normalization function, usually denoted as *q* = Σ*q_i_*, the log of which is the Boltzmann average energy of the system, −⟨*E*⟩*_B_*/*k_B_T*. Statistically, the distribution of the particles is characterized by the multinomial Boltzmann probability density function,

$$\mathrm{Pr}\;\left(n_{1},\;\ldots ,\;n_{m}|N_{\mathrm{total}},\theta_{1},\;\ldots ,\;\theta_{m}\right)=N_{\mathrm{total}}!\prod_{\mathrm{species j}}^{m}\frac{1}{n_{j}!}\theta_{\text{j}}^{n_{\text{j}}}$$

where *n_j_* is the number of particles of species *j*, and there are *N*_total_ particles. In analogy to the macroscopic, the free energy from statistical thermodynamics, an unnormalized mass density for a microscopic state can be defined that is a function of the molecular partition functions *q_i_* instead of the Boltzmann probabilities,

(2) $$\frac{-A\left(n_{1},\;\ldots ,\;n_{m}|N_{T},\;q_{1},\;\ldots ,\;q_{m}\right)}{k_{B}T}=\log\left(N_{\mathrm{total}}!\prod_{j}^{m}\frac{1}{n_{j}!}q_{j}^{n_{j}}\right)$$

For brevity, we will write *A*(*n*_1_, …, *n*_m_ | *N*_r_, *q*_1_, …, *q*_m_) as $A\left(\bar{n}|N_{T},\;\bar{q}\right)$ or simply *A*. The value *A* in Eq. 2 is not a free energy because it is not an average over all possible values for each of the *n_j_*. The relationship between *A* and the probability density of that microscopic state is,

$$-A∕k_{B}T\;=\log\;\mathrm{Pr}\;\left(n_{1},\;\ldots ,\;n_{m}|N_{\mathrm{total}},\;\theta_{1},\;\ldots ,\;\theta_{m}\right)+N_{\mathrm{total}}\cdot \log q$$

or equivalently,

$$\log\mathrm{Pr}\left(n_{1},\ldots ,\;n_{m}|N_{\mathrm{total}},\;\theta_{1},\ldots ,\;\theta_{m}\right)=A∕k_{B}T\;+N_{\mathrm{total}}\cdot \log q$$

Since log *q* = − ⟨*E*⟩*_B_*/*k_B_T*, we have the relationship

(3) $$\begin{array}{l} \begin{array}{rl} -S_{g} & =A∕k_{B}T-N_{T}{\left\langle E\right\rangle }_{B}∕k_{B}T\\ S_{g} & =-\log\mathrm{Pr}\;\left(n_{1},\;\ldots ,\;n_{m}|N_{T},\;\theta_{1},\;\ldots ,\;\theta_{m}\right) \end{array} \end{array}$$

This function on the right hand side is strictly a log likelihood, not an entropy. However, the average log likelihood is an entropy, and in fact is the Gibbs entropy for a system with a fixed number of total particles,

(4) $$\begin{array}{ccc} S_{G} & = & \sum_{\mathrm{microstates}\;J}\mathrm{Pr}\;\left(J\right)\log\mathrm{Pr}\;\left(J\right)\\ & = & \left\langle A\left(\bar{n}|N_{T},\bar{q}\right)\right\rangle -N_{\mathrm{total}}{\left\langle E\right\rangle }_{B} \end{array}$$

where Pr(*J*) is shorthand for Pr(*n*_1_ = *n*_1_(*J*), …, *n_m_*(*J*)|*N*_total_, θ_1_, …, θ*_m_*) and $\left\langle A\left(\bar{n}|N_{T},\bar{q}\right)\right\rangle =𝒜$ is the free energy of the macroscopic state with parameter *N_T_*. Because the Gibbs entropy is an average over microstates, it is the entropy related to macroscopic observations (Jaynes, 1965).

Adding confusion to the definition of entropy is the related microstate relationship,

(5) $$S_{B}=A∕k_{B}T-N_{\mathrm{total}}{\left\langle E∕k_{B}T\right\rangle }_{U}$$

where now ⟨*E*/*k_B_T*⟩*_U_* is the average energy of the microstate under the uniform distribution instead of the Boltzmann distribution. The entropy term is also given by *S* = − Σ*p_j_* log *p_j_* where again the probabilities *p_j_* = *n_j_*/*N*_total_ are from the uniform distribution (Davidson, 1962; Cannon, 2014). The subscript indicates that this is the Boltzmann entropy because it is derived from log*W* where *W* is the multinomial coefficient. This entropy is also sometimes referred to as the configurational entropy (Davidson, 1962). The difference between the Gibbs and Boltzmann entropies of course has to do with intermolecular potentials and microscopic vs. macroscopic perspectives (Jaynes, 1965).

When the total number of particles is not fixed, adjustments need to be made to the equations above. Typically, the adjustment is to remove the normalization of the Boltzmann probabilities in Eq. 1, such that the resulting quantity $e^{-A∕k_{B}T}$ is an unnormalized probability mass function, or an odds of $e^{-A∕k_{B}T}:1.$ The multinomial probability distribution now becomes a multinomial odds distribution, the main difference being that a probability mass function over all of state space sums to 1, while the new multinomial distribution sums to a value >1.

If the total number of particles is allowed to vary due to the system being open, then Eq. 4 gives

$$S_{G}=\left\langle A-N_{\mathrm{total}}\left(J\right)\log q\right\rangle$$

Notice that this definition is different from one common thermodynamic definition of entropy, which defines entropy as the difference between the free energy and the average energy,

$$\begin{array}{llll} S & =\left\langle A\right\rangle -\left\langle E\right\rangle \; & & \;\\ & =\left\langle A\right\rangle -\left\langle N_{\mathrm{total}}\left(J\right)\log q\right\rangle \; & & \; \end{array}$$

Since we know from the triangle inequality, ||log *x* − log *y*|| ≥ ||log *x||* − *||*log *y*||, it follows that *S_G_* ≥ *S*.

For a set of coupled reactions such as,

A⇄B⇄C

a change of the microscopic state from *K* to *J* is described by the likelihood ratio,

(6) $$-\Delta S_{\mathrm{g,JK}}=\log\left(\frac{\mathrm{Pr}\;\left(J\right)}{\mathrm{Pr}\;\left(K\right)}\right),$$

or equivalently,

(7) $$\frac{\mathrm{Pr}\;\left(J\right)}{\mathrm{Pr}\;\left(K\right)}=e^{-\Delta S_{\mathrm{g,JK}}}$$

which has the basic mathematical form of a fluctuation theorem, but in this case is an identity due to the definition of *S_g_* in Eq. 3. If we average over all states *J* and *K* and the system is at equilibrium,

(8) $$\begin{array}{ccc} \left\langle \frac{\mathrm{Pr}\;\left(J\right)}{\mathrm{Pr}\;\left(K\right)}\right\rangle & =\left\langle e^{-\Delta S_{\mathrm{g,JK}}}\right\rangle & \\ & =1 \end{array}$$

where the angular brackets denote an equilibrium average. The average value is unity since the log likelihood of Eq. 6 is zero, on average. Relation 8 simply says, that on average, the system returns to equilibrium. While Eq. 7 is exact for microscopic processes, the challenge in employing it to model time-dependent processes is that the core probabilities available for use in Eq. 1 are stationary Boltzmann probabilities, yet if the individual rates of the reactions vary enough in a system of coupled reactions, the core probabilities will not be Boltzmann probabilities, which are based solely on energy levels of the reactants and products. At equilibrium, Eq. 7 can be used for time-dependent probabilities because of detailed balance –Eq. 8. However, away from equilibrium, Eq. 7 no longer holds because detailed balance no longer exists. Instead, the true probabilities will be a function of the entire energy surface of the system, including the reaction barriers. Fluctuation theorems relate the ratio of these time-dependent probabilities to a function that is related to the time-dependent Δ*S_g_*(*t*), or if ensemble averages are used, the time-dependent Δ*S_G_*(*t*).

For example, at a non-equilibrium steady state the average fluctuations of a system can still be characterized at times without knowing the actual probabilities of each state. Consider the fluctuation away from a steady state *J* to the new state *K* with some transition probability. We know that the system will eventually return to the steady state *J*, we just do not know specifically how. For the most part, a fluctuation away from the steady state will be along the direction of the non-equilibrium driving force. When the system returns to the steady state, an amount of energy will have been dissipated from the system. Note that if the system were to return to the steady state along the same path, no energy would have been dissipated; that is, the average likelihood of returning along the same path is not 1 as in the case for equilibrium (Eq. 8). Thus, fluctuation theorems for non-equilibrium steady state take the form,

(9) $$\Omega ={\left\langle \log\left(\frac{\pi_{\mathrm{KJ}}\left(t\right)}{\pi_{\mathrm{KJ}}\left(t\right)}\right)\right\rangle }_{\mathrm{J,K}}$$

where π*_KJ_* is the probability of trajectory *J* →*K*, and Ω is related to the dissipation of energy due to the non-equilibrium steady state. For instance, the Evans–Searles fluctuation theory relates the time-dependent probabilities to a trajectory-specific dissipation function, Ω(*t*), which is a measure of how far the system is away from detailed balance,

(10) $$\frac{\pi_{\mathrm{KJ}}\left(\Omega \left(t\right)=-q_{D}∕k_{B}T\right)}{\pi_{\mathrm{JK}}\left(\Omega \left(t\right)=q_{D}∕k_{B}T\right)}=e^{-q_{D}∕k_{B}T}$$

If *q_D_* represents the dissipated energy due to the lack of detailed balance, then the odds of regaining that energy through a reversal of the trajectory are exponentially small. One could even think of the RHS of Eq. 10 as representing the energy of a hypothetical particle (a “dissipation”) that has a Boltzmann factor of $e^{-q_{D}∕k_{B}T}.$ Recent developments in fluctuation theories (reviewed by Sevick et al., 2008; Seifert, 2012) in the last two decades have pushed the envelope into the far from equilibrium domain. Many biochemical reactions are in this domain.

### Entropy production

When the time-dependent flux of material through reactions can be determined, the entropy production rate can be defined in several related ways (Oster et al., 1973; Ge et al., 2006; Ge and Qian, 2010). Using Eq. 6, the microscopic entropy production can be defined for a reaction *i* in the +direction as,

$$\mathrm{microscopic entropy production rate}=J_{i+}\Delta S_{\mathrm{g,i}}$$

and the net entropy production through the reaction is *J*_i,net_Δ*S*_g,i_, where *J*_i,net_ = *J_i+_* − *J_i_*_−_. Taking the ratio of the entropy production due to the forward and the reverse reaction, the odds of entropy being produced at reaction *i* are,

(11) $$\begin{array}{ccc} O\left(\Delta S_{\mathrm{g,i}}\right) & =\frac{J_{i+}\cdot \Delta S_{\mathrm{g,i}}}{J_{i-}\cdot \Delta S_{\mathrm{g,i}}} & \\ & =\frac{J_{i+}}{J_{i-}} \end{array}$$

Although the ratio of the forward and reverse flux gives us the odds of thermodynamic entropy production, the ratio itself cannot tell us the value of the thermodynamic entropy change or even if the entropy change is positive or negative; in coupled systems the flux through any specific reaction is not deterministically related to the entropy or free energy change of that reaction. The second law of thermodynamics only tells us that for macroscopic processes, the entropy must always increase; the second law does not address what might be happening on the microscopic level in individual reactions. This is an important aspect of stochastic systems: even though a reaction has a free energy change above zero or equivalently an odds below one, it can still occur given enough time. For example, if a set of coupled reactions has a large enough overall favorable change in free energy, an individual reaction can have a net positive flux even if the reaction free energy is unfavorable. Flux is an emergent property of the entire system. However, as indicated by the fluctuation theorems, the less likely the reaction, the less likely it will have a net flux in the direction of decreasing entropy change.

Several studies have asserted that the relationship between flux and free energy is Δ*G* = −*RT*log(*J*_+_/*J*_−_). This relationship was originally proposed in discussions of reversible systems and discussed in the context of deterministic kinetics (Beard and Qian, 2007). For coupled, stochastic non-equilibrium reactions, the relationship is strictly speaking an assumption. However, it is reasonable to expect in the vast majority of situations that Δ*G* and −*RT*log(*J*_+_/*J*_−_) are concordant. The relationship can be used to gain insight if used carefully. For instance, Noor et al. (2014) have used the assumption as a framework for evaluating flux statistics at individual reactions. They correctly pointed out that reactions near equilibrium act as kinetic bottlenecks in pathways that are overall far from equilibrium. This is a valid use of the assumption in that reactions at equilibrium in an otherwise nonequilibrium system are those for which the relation is approximately correct even for stochastic systems.

So far the question of how to find the steady states has been left open. A steady state could be determined by the textbook approach of solving the set of differential rate equations. However, for biological systems the required rate parameters are rarely available. In principle, a steady state can be defined based on experimental measurement of all relevant chemical species, which can be used to define the chemical potential of each species. While this task is much easier than determining all the appropriate rate constants, it is still formidable. Yet, significant progress is being made (Bennett et al., 2009).

Alternatively, one can assume that the steady state is one that corresponds to an optimal thermodynamic process. A thermodynamically optimal process is one in which a maximal amount of energy can be extracted from the environment with a minimal amount of dissipation of heat (Sivak and Crooks, 2012). Equivalently, a thermodynamically optimal path is one that requires the least work to maintain the steady state. In either case, the thermodynamically optimal steady state can be found by maximizing a steady state version of Eq. 4 in which the Gibbs entropy *S_G_* in a state space neighborhood Γ measures the probability density of states reachable from an initial state 𝕊 due to a series of *Z* reactions involving a change of state δ𝕊_*i*_ (Cannon, 2014),

(12) $$S_{g}\left(\Gamma \left(S\right)\right)=-\sum_{\mathrm{Rxn}\;i=1}^{Z}\mathrm{Pr}\left(S_{i-1}+\delta \;S_{i}\right)\log\mathrm{Pr}\left(S_{i-1}+\delta \;S_{i}\right)$$

In a system moving toward equilibrium through a trajectory of *Z* reactions, the state entropy increases as the system stabilizes, and reaches a maximum at equilibrium since equilibrium requires that each respective reaction is equally likely. In a non-equilibrium system, the neighborhood Γ is a reaction path and Eq. 12 is the path entropy described by Dewar, from which the fluctuation theorem, the selection principle of maximum entropy production, and self-organized criticality can be derived (Dewar, 2003). An analogous Gibbs entropy can be defined by averaging *S_g_*[Γ(𝕊)] over many trajectories such that *S_G_*[Γ(𝕊)] = ⟨*S_g_*[Γ(𝕊)]⟩. If the entropy change from equilibrium is $\Delta S_{G}\left(\Gamma \left(S\right)\right)S_{G}^{0}-S_{G}\left(\Gamma \left(S\right)\right),$ then the rate of production of thermodynamic entropy can then be defined as,

$$\mathrm{thermodynamic}\;\mathrm{entropy}\;\mathrm{production}\;\mathrm{rate}=J_{\mathrm{net}}\left(\Gamma \right)\Delta S_{G}\left(\Gamma \left(S\right)\right)$$

While its likely that no individual organism is at the apex of thermodynamic optimality, it is also likely, as discussed in the section “Introduction,” that natural selection is at some fundamental level based on filtering out individuals that are thermodynamically inefficient such that too little energy is extracted from the environment or too much of the extracted energy is simply dissipated back to the environment; such a system would not be able to channel sufficient energy into growth to compete against more efficient individuals. In this scenario of natural selection, thermodynamically optimal steady states would serve as useful models.

#### Applications

Beyond atomistic simulations, the application of statistical thermodynamics and fluctuation theory to biological systems is truly a frontier. To date, applications are mostly in the physics literature and include (but are not limited to) the study of molecular motors, mostly ATP synthase (Andrieux and Gaspard, 2006; Hayashi et al., 2010; Zimmermann and Seifert, 2012), small metabolic networks (Cannon, 2014), bifurcation dynamics of reaction pathways (Xiao et al., 2009), and models of the response of bacteria to changes in the environment (Barato et al., 2014). These examples were chosen to represent a hierarchy of scales in which statistical thermodynamic simulations have been applied to biology. Because the dynamics of each system is represented using different equations, it is not possible to describe in detail the form of the fluctuation theorem used other than to say that all are in some way represented by Eq. 9, except where noted. Details on the theorems are best obtained from the original literature. Below, we briefly summarize the findings for this representative selection from the literature.

### Single molecule dynamics of ATPase F1 rotary motor

The F_0_F_1_–ATP synthase complex is an example of a highly non-equilibrium nanomotor. The rotary motor of F_0_F_1_–ATP synthase is powered by proton flow across a gradient producing a free energy difference of 10–20 kJ/mol of protons. This free energy difference is significantly greater than the ambient energy at room temperature of about 2.45 kJ/mol. The motor operates over a large range of scales; rate constants for the various processes making up the motor vary over 12 orders of magnitude. Andrieux and Gaspard used fluctuation theory and generating functions to evaluate statistical distributions of mean rotation of the F_1_ rotor, the dissipated work, and the probability flux across the system (Andrieux and Gaspard, 2006). The analysis showed that the ATPase motor has a highly non-linear response to chemical fuel: the mean velocity of the F_1_ rotor as a function of the thermodynamic driving force is a sigmoid-like curve. Despite the microscopic nature of the motor, the operation of the motor is highly robust in this non-linear regime: successive rotations are statistically correlated and remain essentially unaffected by the fluctuations. Nevertheless, it was shown that the fluctuation theorem held even in the highly non-linear regime.

### Multiple molecules: Pathway bifurcation dynamics of a circadian clock

When multiple reactions are coupled, non-intuitive behavior can result. The Lotka–Voltera oscillator and the Brusselator are famous early examples where feedback or feed-forward interactions control the oscillatory behavior. At the cell level, an important oscillatory phenomenon is the circadian clock of organisms as diverse as fruit flies and fungi. In the circadian clock negative feedback controls, the rate of transcription and translation of specific proteins that in turn dictate the cellular circadian oscillation cycle (Dunlap, 1999).

Using a stochastic thermodynamics approach pioneered by Seifert and colleagues, Xiao et al. (2009) used a chemical Langevin equation to evaluate dynamic bifurcations that occur in the circadian clock. An explicit expression for the mean entropy production in the stationary state was formulated based on available kinetic data. On either side of the bifurcation in the circadian dynamics, the shape of the distribution of the entropy production was similar and highly skewed such that the probability of observing dynamics with negative entropy production was quite small. Thus, like the F1 motor of ATP synthase, the operation of the molecular circadian clock studied by Xiao et al. is robust despite the stochastic nature of small systems.

Although the time dependence of the entropy production in the fluctuation theorem used in this study ultimately came from rate constants, the approach demonstrated that statistical thermodynamic simulations are capable of producing similar bifurcation dynamics as stochastic kinetic simulations. Understanding the entropy production rates of metabolism is important for quantitating the capacity for organisms to adapt to their changing environment, which is discussed next.

### Cellular information processing and adaptation

Philosophically, one can adopt either of two opposing perspectives about the relationship between simple biological systems such as bacteria and their environment. One can take the perspective that cells make decisions based on their external environment, which is the most discussed perspective in the literature, or one can take the perspective that the external environment determines cellular response. While the former perspective imbues autonomy to the cell, the latter perspective takes the view that regulation is ultimately a function of the external environment. Who is driving – the cell or the environment? While the former perspective is correct on short time periods such as the diurnal cycles, the latter perspective is more correct on longer time periods over which the cell has adapted and evolved.

Barato et al. (2014), evaluated models of how much information cells can extract from their environment based on their thermodynamic efficiency. Although Barato et al. use the metaphor of learning for the ability to extract information, one is equally justified in using the concept of self-organization. The study found that the degree to which a cell can self-organize in response to the environment is bounded by the thermodynamic entropy production rate. A bacterium in a slowly changing environment dissipates much more energy than it harnesses for the purpose of self-organization. That is, the bacterium, once organized to respond to a particular environment, has a limited ability to further harness energy from the environment for further adaptation.

Although Barato et al. (2014) used quite simple physical models to generate hypotheses, clearly coupling this framework with more extensive thermodynamic models of metabolism has the potential to provide insight into how cells respond internally to changes in environmental driving forces on both short time scales and longer evolutionary time scales. However, modeling efforts will require more sophisticated models of metabolism in order to understand the multitude of paths that cell behavior can take. Next, early efforts that have been taken to expand the application of statistical thermodynamics to more detailed metabolic models are discussed.

### Detailed metabolic models

The models and systems discussed above are small systems compared to the metabolism of even the smallest bacterium. Can statistical thermodynamics and fluctuation theories also be applied to more extensive biological systems such as genome-scale models of metabolism? The issue mostly pertains to whether sufficient parameters can be estimated. Large-scale estimates of thermodynamic parameters are available from sources such as the Biochemical Reactions Thermodynamics Database at University of Michigan (Li et al., 2011), the Thermodynamics of Enzyme-Catalyzed Reactions Database at NIST (Goldberg et al., 2004), and the eQuilibrator web server (Flamholz et al., 2012).

We have been developing such an approach and to-date have applied it to relatively small metabolic pathways of various bacteria (Cannon, 2014). In these initial studies, the reactions rates are assumed to be proportional to the thermodynamic driving force of the reaction, which is quantified by a probability of a reaction in a Markov model based on Eq. 7.

Initial studies have focused on the tricarboxylic acid (TCA) cycles of bacteria. These cycles are central to the metabolism of most organisms and may be as close to a universal pathway as there is (Smith and Morowitz, 2004). TCA cycles are capable of consuming acetyl-CoA to either produce high energy compounds necessary for cell function (e.g., ATP, NADPH) or carbon backbones that serve as synthetic precursors for many reactions of secondary metabolism and amino acid and nucleic acid synthesis.

**Figure 1:**
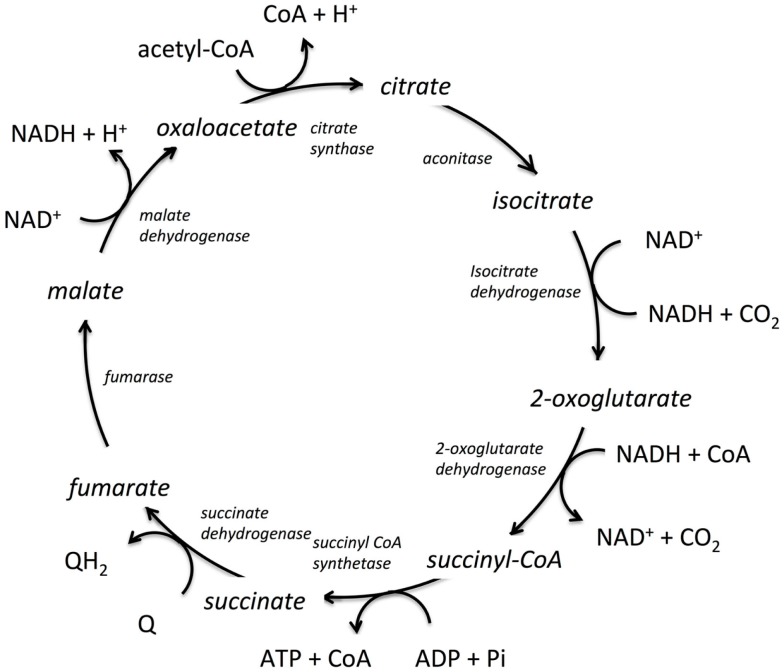
**The tricarboxylic acid cycle (TCA) from *E. coli***. The enzymes catalyzing the reactions are shown in italics, the co-factors are shown tangentially to each respective reaction, and the reaction intermediates are shown in line with the cyclic reaction arrows indicating direction of the cycle for *E. coli*. Q and QH_2_ are electron acceptor/donator pairs and are entry points to the electron transfer chain.

**Figure 2:**
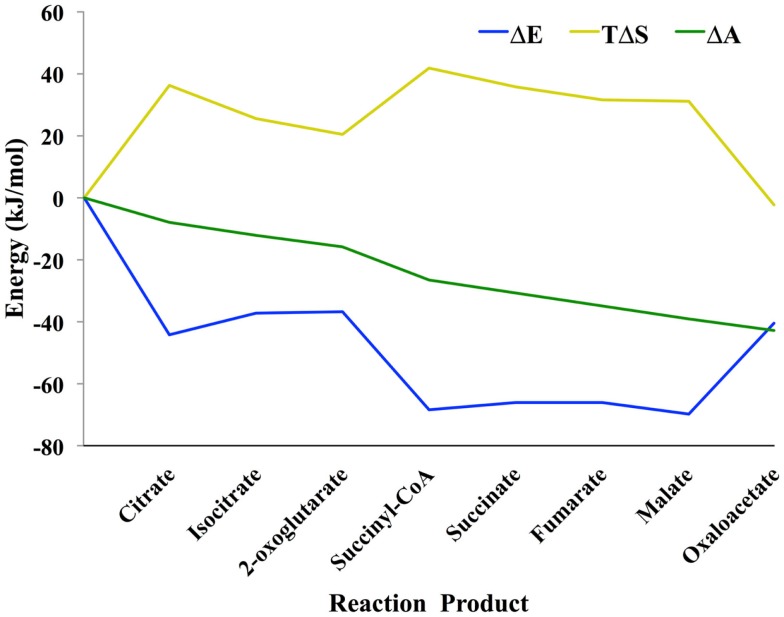
**Thermodynamic profile of the TCA cycle from *E. coli* (Cannon, 2014)**. Eq. 4 was used to calculate the change in entropy Δ*S*, energy Δ*E*, and the log of the (unnormalized) mass density Δ𝒜. Because the probability mass density consists of a combinatorial coefficient that is represented by the entropy term and an energy-based (Boltzmann) probability, there is energy–entropy compensation throughout the cycle. Δ𝒜 changes smoothly across the reaction pathway indicating that the concentrations of the metabolites are close to optimal, likely because the concentrations were taken from an experimental measurement of *E. coli* metabolite levels.

Shown in Figure 1 is the TCA cycle of *E. coli* and in Figure 2 is the free energy, energy, and entropy profiles under metabolic conditions observed for exponential growth on glucose (Bennett et al., 2009). The cycle was simulated using statistical thermodynamics formulation of a Markov model based on a local equilibrium assumption (Cannon, 2014). As one proceeds from acetyl-CoA clockwise around the cycle to oxaloacetic acid, the free energy change across the reactions (Figure 2) varies smoothly, as one would expect from a maximum entropy perspective (Eq. 12). However, the change for the conversion of oxaloacetate and acetyl-CoA to citrate catalyzed by citrate synthase and the change for the conversion of 2-oxoglutarate to succinyl CoA catalyzed by 2-oxoglutarate dehydrogenase are somewhat abrupt compared to changes at the other reactions of the cycle. The reason for this is that the cofactor concentrations, which serve as boundary conditions, are held fixed at values that prevent the system from relaxing further. As a result, the system is not quite thermodynamically optimal – the entropy defined by Eq. 12 is not quite maximal compared to the value that would be obtained if each reaction was equally likely.

Clearly, information about the thermodynamics of biosynthetic pathways is important for engineering metabolism to overproduce target compounds such as reduced carbon compounds for biofuels. While much attention has been directed at redirecting carbon flow by knocking out pathways competing for precursors, less attention has been directed at engineering redox pairs such as NADH:NAD^+^ levels that would thermodynamically drive these reactions. Likewise, much attention has focused on the use of riboswitches to up-regulate the production of enzymes involved in the biosynthesis of target compounds (Wittmann and Suess, 2012), but switching on the catalytic machinery to synthesize a compound is not useful unless the thermodynamics of the pathway are favorable. Modeling metabolic systems thermodynamically would be of enormous value for metabolic engineering.

**Figure 3:**
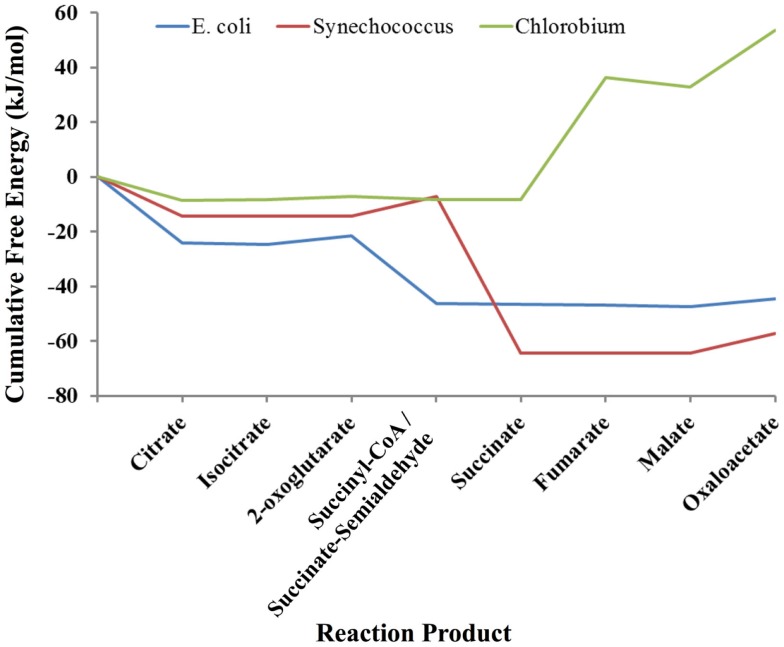
**Comparison of the thermodynamic profiles of the TCA cycles of *E. coli, Synechococcus sp*. *PCC 7002 and Chlorobium tepidum***. The free energy profile of the TCA cycle for each organism reflects its environmental niche (see Discussion).

As an example of the potential use of statistical thermodynamics for both engineering and understand organisms in the context of their natural habitats, we compared three different versions of the TCA cycle used in three very different ecological niches: a typical heterotrophic TCA cycle from *E. coli* involved in extracting energy and biosynthetic precursors from glucose; the cyanobacterial TCA cycle of *Synechococcus* sp. PCC 7002, which is required to produce biosynthetic precursors despite already high levels of ATP from photosynthesis; and the TCA cycle of *Chlorobium tepidum*, a green sulfur bacteria that also must produce biosynthetic precursors in the presence of photosynthesis and simultaneously fix CO_2_, which it does by running the TCA cycle in the reductive direction_._ As above, each TCA cycle was simulated using a Markov model based on a local equilibrium assumption. The free energy profiles for these organisms are shown in Figure 3. Clearly, each pathway is very different thermodynamically. The cycles for *E. coli* and Synechococcus have similar profiles except for the conversion of 2-oxoglutarate to succinate. In the *E. coli* TCA cycle, this reaction has ATP as a product. *Synechococcus* and other cyanobacteria cannot use the same reaction for converting 2-oxoglutarate to succinate cycle because their cycles must operate in an environment in which ATP concentrations are quite high due to concomitant photosynthesis. Instead, the cyanobacteria use a TCA cycle that employs a ferredoxin coenzyme for this conversion, and thus high levels of ATP do not retard the production of succinate and other carbon compounds that are necessary for growth. The free energy profile of the TCA cycle for *Chlorobium* is very different from both the *E. coli* and *Synechococcus* cycles. Instead of having a highly favorable free energy profile for operation in the oxidative direction (citrate → oxaloacetate), the free energy changes are highly unfavorable. The TCA cycle of *Chlorobium* and other green sulfur bacteria, in fact, runs in the opposite direction (oxaloactetate → citrate), and these organisms use the cycle to fix CO_2_ and produce acetyl-CoA. Not only does a thermodynamic model allow us to understand each organism in its environment, but clearly designing an optimal pathway for metabolic engineering using statistical thermodynamics would be very useful.

In comparing the free energy profiles for *E. coli* in Figures 2 and 3, it is clear that they differ significantly. In Figure 2, the free energy profile changes relatively smoothly as one traverses the cycle, while in Figure 3 the free energy profile changes abruptly at times. The reason for these differences has to do with the conditions used in the respective simulations. In Figure 2, the simulations used the published experimentally measured values for *E. coli* (Bennett et al., 2009). In the latter case, the count of each intermediate in the cycle was initially set to ~20 μm each instead of using the experimental published values for *E. coli* (Bennett et al., 2009), which otherwise might bias the comparison between the three organisms. Although each cycle is materially open in that two carbons come in as acetyl-CoA and carbons leave as CO_2_, the total of the number of intermediates is fixed by the stoichiometry of the overall reaction for completion of the cycle. For *E. coli*, the overall stoichiometry is,

$$\begin{array}{llll} & \mathrm{Acetyl -CoA}+\mathrm{ADP}+3\mathrm{NA}D^{+}+P_{i}+Q+2H_{2}O\; & & \;\\ & \;⇌\mathrm{CoA}+\mathrm{ATP}+3\mathrm{NADH}+2CO_{2}+QH_{2},\; & & \; \end{array}$$

where Q and QH_2_ represent an oxidized and reduced electron carriers, respectively. Although the cycles are open, the sum of the count of all intermediates will only vary by ±1.

**Figure 4:**
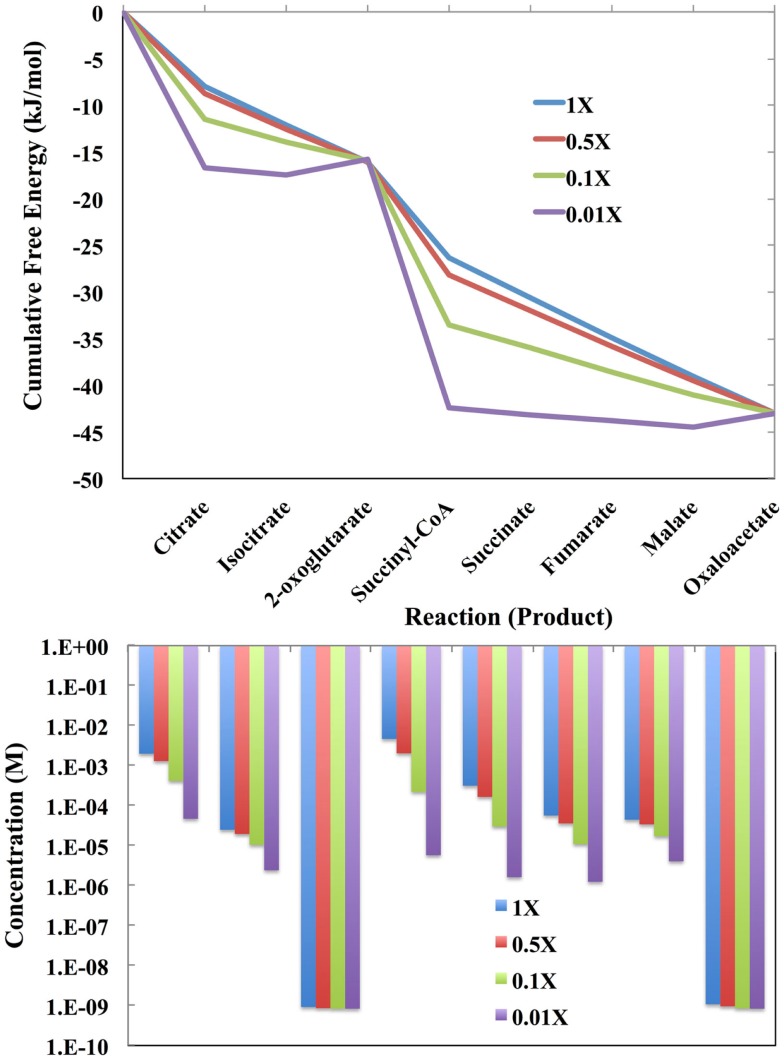
**(Top) The cumulative free energy profile of the *E*. *coli* TCA cycle as a function of the total concentration of the reaction intermediates**. Although carbon can enter the cycle as acetyl-coa and leave as CO_2_, the total number of intermediates is constrained by the overall reaction (see text). The concentrations used are 1-fold, 0.1-fold, 0.01-fold, and 0.001-fold of those reported by Bennett et al. (2009) for exponential growth on glucose. (Bottom) The distribution of reaction intermediates as a function of total concentration.

The free energy profiles of the *E. coli* TCA cycle as a function of the total concentration of the intermediates are shown at the top of Figure 4. The total concentration values are 1.0-fold, 0.1-fold, 0.01-fold, and 0.001-fold of the values reported by Bennett et al. (2009). If there are only a few total intermediates, then these will be transformed into the metabolites with lowest chemical potentials, which in the case of the *E. coli* TCA cycle are citrate and succinyl CoA. At very low levels of intermediates, the cycle will not operate and citrate and succinyl CoA will simply pool. For the lowest level of intermediates, there will be flux through the entire cycle only over relatively long time periods.

As the total number of metabolic intermediates is raised, the number of citrate and succinyl CoA molecules increase, as shown in Figure 4 (bottom). Eventually, product builds up as well with a concomitant increase in the free energies of reactions producing citrate and succinyl CoA. Meanwhile, the increase in citrate decreases the free energy for the citrate to isocitrate reaction, and likewise, the increase in succinyl CoA decreases the free energy for the succinyl CoA to succinate reaction.

Eventually, metabolite levels build up to the point where all reactions become equally likely in agreement with Eq. 12. This is thermodynamically the most optimal since the state entropy (Eq. 12) has been maximized with respect to the non-equilibrium boundary conditions.

However, for the cell there is also a thermodynamic penalty to obtain this configuration. In order to handle a greater number of reactants, the enzymatic load on the cell must likewise increase. The self-organized structures needed to dissipate energy rapidly (or store the harvested energy for growth) must be paid for by the non-equilibrium driving forces.

Enzymes catalyzing reactions far from equilibrium will need to increase the least since material flow is unidirectional. This is clearly the case for the enzyme-catalyzed reactions for transformation of oxaloacetate to citrate and 2-oxoglutarate to succinate: as the total metabolite pool increases, the concentrations of the reactants oxaloacetate and 2-oxoglutarate do not change markedly.

If enzymes near equilibrium are expressed at a level just sufficient to catalyze its current load, then increasing the total pool of metabolites may require increased expression of these enzymes. However, these reactions are not likely to remain at equilibrium. This is apparent in Figure 4 (top) in which the last four enzyme-catalyzed reactions of the TCA cycle transforming succinyl CoA to oxaloacetate, are close to equilibrium when the total pool of metabolites is 0.001-fold of the values reported by Bennett et al. (2009). As the total metabolite pool grows, the reactions do not remain at equilibrium.

When metabolite levels are greater than the respective Michaelis constant (*K*_M_), then enzyme levels need to increase in order to maintain a steady state. This is the situation described by Flamholz et al. (2013). That enzymes catalyzing reactions far from equilibrium do not increase significantly has been experimentally observed; the degree to which enzyme expression will need to increase for reactions near equilibrium will be situation dependent but generally will need to increase with increased flux (Hochachka et al., 1998).

Moreover, if the turnover rates for the enzymes in the pathway differ dramatically, then there must also be a differential level of expression of the enzymes in the pathways. It would make sense for the organism to have high intrinsic enzyme turnover rates for costly enzymes, either those that have many amino acids or require high energy co-factors, such that the thermodynamic cost to the cell can be minimized (Flamholz et al., 2013).

Considering Figure 4 (top), the data reported by Bennett et al. (2009), implies that the TCA cycle of the laboratory strain of *E. coli* is operating near optimal efficiency with regard to Eq. 12 during exponential growth on glucose. In Lotka’s words, “the struggle for existence, the advantage must go to those organisms whose energy-capturing devices are most efficient in directing available energy into channels favorable to the preservation of the species.”

How close are biological systems to optimal efficiency? There appear to be situations when this ideal is not achieved. For example, if glycolysis were left unchecked such that each reaction were equally likely thermodynamically, then the large free energy change for conversion of fructose 6-phosphate to fructose 1,6-bisphosphate would result in cellular concentrations of fructose 1,6-bisphosphate several orders of magnitude higher than is observed, which would most likely have detrimental affects on the cell. In fact, the enzyme catalyzing this step is highly regulated to prevent overproduction of fructose 1,6-bisphosphate. The regulation can be regarded as a self-organized and emergent property of the pathway, and one that is necessary for the organism to remain viable. Considering the framework for adaption laid out by Barato et al. (2014), this would imply that for *E. coli* species that are adapted to growth on high levels of glucose, there are very little opportunities for learning alternative ways of regulating this enzyme, or conversely, that the regulatory circuit is evolutionarily stable in this regard.

#### Future Directions

Determining a rate constant for an enzyme of interest is a straight-forward task if the reactant or product has a distinct spectroscopic signature. However, scaling the process up to obtain all of the rate constants necessary for large-scale simulations of metabolism of any specific organism is simply not feasible. Mixing and matching rate constants from orthologous enzymes from different species can result in incorrect energetics, unless one constrains the rate constants to match the equilibrium constant for the same reaction. Moreover, *ad hoc* adjustment of a rate constant to obtain the correct equilibrium constant is likely not better than assuming rates are proportional to the thermodynamic driving force. As a result of the difficulty in obtaining rate constants, constraint-based flux models have been the method of choice for large-scale modeling of biological processes such as metabolism. However, constraint-based methods at best use the thermodynamic constraints to narrow down the solution space. Unfortunately, this limits the predictive power of these approaches.

Several promising and fundamentally sound approaches that include proper thermodynamics have been proposed to move beyond constraint-based flux modeling. One approach is to model systems using mass action kinetics for those reactions for which rate parameters are available, and to use constraint-based flux modeling of other reactions (Chowdhury et al., 2014). In this case, the fluxes modeled using mass action kinetics limit the range of fluxes that are possible for those reactions modeled with constraint-based flux modeling.

A second approach is to use available kinetic parameters where one can, and then infer the remaining parameters based on prior knowledge, including balancing rate parameters to ensure that the correct thermodynamics are obtained (Stanford et al., 2013). An alternative is to reduce the kinetic complexity of the rate equation of each reaction-based analysis of the reaction likelihood as a function of the net flux of the reaction (Canelas et al., 2011). For some reactions, the rate parameters can be eliminated altogether and replaced by the thermodynamic likelihood of the reaction without compromising the fidelity of the model.

Finally, if one knows the reaction directionality, such as from an experimentally based metabolic flux analysis, then a set of feasible metabolite concentrations and reaction free energies can be determined using optimization methods (De Martino et al., 2012). The ability to map out the energy landscape of metabolism could be very powerful and could inform us on whether the conjectures by Lotka, Odum, and others about natural selection discussed in the section “Introduction” are correct. The criteria used by De Martino et al. may actually be too stringent in that the optimization constraints required that the entropy production for each reaction be positive. As indicated in the section “Discussion” around Eq. 11, the second law only requires that the entropy production for the overall macroscopic process be positive. An individual reaction may have a positive flux and also a positive free energy change, but the chance of such an event decreases exponentially with increases in the free energy (Evans and Searles, 1994). The analysis requires the input of flux configurations or reaction directionality. However, this is where fluctuation theories can play a role if they can provide flux values as well.

The use of detailed fluctuation theorems will depend on whether theorems can be developed for non-equilibrium steady states that do not use rate constants and are instead based on chemical potentials and thermodynamic driving forces. If so, then one can set the chemical potentials based (ideally) on metabolomics measurements and carry out large-scale simulations of metabolism that would be identical to kinetic simulations based on rate constants. Experimentally measuring metabolite concentrations is an emerging area of great interest. Key to making the measurements useful for interpretation and modeling is reducing the uncertainty that the measured values reflect *in vivo* concentrations (Noack and Wiechert, 2014).

An alternative statistical thermodynamic approach is to model the process as thermodynamically optimal in which the rates are proportional to the thermodynamic driving force. In a thermodynamically optimal process, the maximum amount of energy is extracted from the environment with a minimal amount of dissipation of heat (Sivak and Crooks, 2012). A model based on this assumption would be roughly consistent with the historical perspectives of the physical basis of biological systems. An analogous approach has been used to analyze metabolomics data, in which the free energies of reactions are minimized with respect to available metabolomics data in order to infer sites of enzyme regulation (Kummel et al., 2006).

As mentioned above, a challenge to using simulations based on statistical thermodynamics is determining accurate standard free energies of reaction or formation of each metabolite. Standard free energies based on group contribution methods are available *en masse* (Jankowski et al., 2008; Noor et al., 2013), but group contribution methods can be inaccurate at times. One must be careful when estimating a standard reaction free energy from group contribution estimates of standard formation free energies in that the errors in estimates are additive; one must ensure when taking the difference between two chemical species that any approximations used for group energies cancel out. The use of electronic structure calculations with an appropriate solvent model is an attractive alternative for determining standard free energies and chemical potentials. Such calculations have been done on a large scale for chlorinated hydrocarbons (Bylaska, 2006) and it is feasible to carry these out for many metabolites. Larger molecules from secondary metabolism, such as those from plants, may present a challenge in that they may have multiple minima that contribute to their free energy of solvation.

## Conflict of Interest Statement

The author declares that the research was conducted in the absence of any commercial or financial relationships that could be construed as a potential conflict of interest.

## References

[B1] Andrieux D. Gaspard P. (2006). Fluctuation theorems and the nonequilibrium thermodynamics of molecular motors. Phys. Rev. E 74, 011906. 10.1103/PhysRevE.74.011906 16907126

[B2] Barato A. C. Hartich D. Seifert U. (2014). Efficiency of cellular information processing. New J. Phys. 16, 103024 10.1088/1367-2630/16/10/103024

[B3] Beard D. A. Qian H. (2007). Relationship between thermodynamic driving force and one-way fluxes in reversible processes. PLoS ONE 2:e144. 10.1371/journal.pone.0000144 17206279 PMC1764038

[B4] Bennett B. D. Kimball E. H. Gao M. Osterhout R. Van Dien S. J. Rabinowitz J. D. (2009). Absolute metabolite concentrations and implied enzyme active site occupancy in *Escherichia coli*. Nat. Chem. Biol. 5, 593–599. 10.1038/nchembio.186 19561621 PMC2754216

[B5] Bylaska E. J. (2006). Estimating the thermodynamics and kinetics of chlorinated hydrocarbon degradation. Theor. Chem. Acc. 116, 281–296. 10.1007/s00214-005-0042-83097319

[B6] Canelas A. B. Ras C. Ten Pierick A. Van Gulik W. M. Heijnen J. J. (2011). An in vivo data-driven framework for classification and quantification of enzyme kinetics and determination of apparent thermodynamic data. Metab. Eng. 13, 294–306. 10.1016/j.ymben.2011.02.005 21354323

[B7] Cannon W. R. (2014). Simulating metabolism with statistical thermodynamics. PLoS ONE 9:e103582. 10.1371/journal.pone.0103582 25089525 PMC4121145

[B8] Chowdhury A. Zomorrodi A. R. Maranas C. D. (2014). k-OptForce: integrating kinetics with flux balance analysis for strain design. PLoS Comput. Biol. 10:e1003487. 10.1371/journal.pcbi.1003487 24586136 PMC3930495

[B9] Davidson N. (1962). Statistical Mechanics. New York, NY: McGraw.

[B10] De Martino D. Figliuzzi M. De Martino A. Marinari E. (2012). A scalable algorithm to explore the Gibbs energy landscape of genome-scale metabolic networks. PLoS Comput. Biol. 8:e1002562. 10.1371/journal.pcbi.1002562 22737065 PMC3380848

[B11] Dewar R. (2003). Information theory explanation of the fluctuation theorem, maximum entropy production and self-organized criticality in non-equilibrium stationary states. J. Phys. A Math. Gen. 36, 631–641 10.1088/0305-4470/36/3/303

[B12] Dewar R. (2005). “Maximum entropy production and non-equilibrium statistical mechanics,” in Non-equilibrium Thermodynamics and the Production of Entropy, eds Kleidon A. Lorenz R. (Berlin: Springer), 41–55.

[B13] Dunlap J. C. (1999). Molecular bases for circadian clocks. Cell 96, 271–290 10.1016/S0092-8674(00)80566-8 9988221

[B14] Evans D. J. Searles D. J. (1994). Equilibrium microstates which generate 2nd law violating steady-states. Phys. Rev. E 50, 1645–1648 10.1103/PhysRevE.50.1645 9962139

[B15] Flamholz A. Noor E. Bar-Even A. Liebermeister W. Milo R. (2013). Glycolytic strategy as a tradeoff between energy yield and protein cost. Proc. Natl. Acad. Sci. U.S.A. 110, 10039–10044. 10.1073/pnas.1215283110 23630264 PMC3683749

[B16] Flamholz A. Noor E. Bar-Even A. Milo R. (2012). eQuilibrator – the biochemical thermodynamics calculator. Nucleic Acids Res. 40, D770–D775. 10.1093/nar/gkr874 22064852 PMC3245061

[B17] Ge H. Jiang D. Q. Qian M. (2006). Reversibility and entropy production of inhomogeneous Markov chains. J. Appl. Probab. 43, 1028–1043 10.1239/jap/1165505205

[B18] Ge H. Qian H. (2010). Physical origins of entropy production, free energy dissipation, and their mathematical representations. Phys. Rev. E 81, 051133. 10.1103/PhysRevE.81.051133 20866211

[B19] Goldberg R. N. Tewari Y. B. Bhat T. N. (2004). Thermodynamics of enzyme-catalyzed reactions – a database for quantitative biochemistry. Bioinformatics 20, 2874–2877. 10.1093/bioinformatics/bth314 15145806

[B20] Harris R. J. Schutz G. M. (2007). Fluctuation theorems for stochastic dynamics. J. Stat. Mech. 2007, 07020 10.1088/1742-5468/2007/07/P07020

[B21] Hayashi K. Ueno H. Iino R. Noji H. (2010). Fluctuation theorem applied to F1-ATPase. Biophys. J. 98, 633A–633A. 10.1016/j.bpj.2009.12.3466 20867140

[B22] Hochachka P. W. Mcclelland G. B. Burness G. P. Staples J. F. Suarez R. K. (1998). Integrating metabolic pathway fluxes with gene-to-enzyme expression rates. Comp. Biochem. Physiol. B 120, 17–26 10.1016/S0305-0491(98)00019-4

[B23] Jankowski M. D. Henry C. S. Broadbelt L. J. Hatzimanikatis V. (2008). Group contribution method for thermodynamic analysis of complex metabolic networks. Biophys. J. 95, 1487–1499. 10.1529/biophysj.107.124784 18645197 PMC2479599

[B24] Jaynes E. T. (1965). Gibbs vs. Boltzmann entropies. Am. J. Phys. 33, 391–398 10.1119/1.1971557

[B25] Kleidon A. Malhi Y. Cox P. M. (2010). Maximum entropy production in environmental and ecological systems. Philos. Trans. R. Soc. Lond. B Biol. Sci. 365, 1297–1302. 10.1098/rstb.2010.0018 20368247 PMC2871911

[B26] Kummel A. Panke S. Heinemann M. (2006). Putative regulatory sites unraveled by network-embedded thermodynamic analysis of metabolome data. Mol. Syst. Biol. 2, 2006.0034. 10.1038/msb4100074 16788595 PMC1681506

[B27] Li X. Wu F. Qi F. Beard D. A. (2011). A database of thermodynamic properties of the reactions of glycolysis, the tricarboxylic acid cycle, and the pentose phosphate pathway. Database (Oxford) 2011, bar005. 10.1093/database/bar005 21482578 PMC3077827

[B28] Lotka A. J. (1922a). Contribution to the energetics of evolution. Proc. Natl. Acad. Sci. U.S.A. 8, 147–151 10.1073/pnas.8.6.147 16576642 PMC1085052

[B29] Lotka A. J. (1922b). Natural selection as a physical principle. Proc. Natl. Acad. Sci. U.S.A. 8, 151–154 10.1073/pnas.8.6.151 16576643 PMC1085053

[B30] Noack S. Wiechert W. (2014). Quantitative metabolomics: a phantom? Trends Biotechnol. 32, 238–244. 10.1016/j.tibtech.2014.03.006 24708998

[B31] Noor E. Bar-Even A. Flamholz A. Reznik E. Liebermeister W. Milo R. (2014). Pathway thermodynamics highlights kinetic obstacles in central metabolism. PLoS Comput. Biol. 10:e1003483. 10.1371/journal.pcbi.1003483 24586134 PMC3930492

[B32] Noor E. Haraldsdottir H. S. Milo R. Fleming R. M. (2013). Consistent estimation of Gibbs energy using component contributions. PLoS Comput. Biol. 9:e1003098. 10.1371/journal.pcbi.1003098 23874165 PMC3708888

[B33] Odum H. T. Pinkerton R. T. (1955). Time’s speed regulator: the optimum efficiency for maximum power output in physical and biological systems. Am. Sci. 43, 331–343.

[B34] Oster G. F. Perelson A. S. Katchals A. (1973). Network thermodynamics – dynamic modeling of biophysical systems. Q. Rev. Biophys. 6, 1–134 10.1017/S0033583500000081 4576440

[B35] Prigogine I. (1978). Time, structure, and fluctuations. Science 201, 777–785 10.1126/science.201.4358.777 17738519

[B36] Schrödinger E. (1945). What is Life? The Physical Aspect of the Living Cell. Cambridge: The University Press.

[B37] Seifert U. (2012). Stochastic thermodynamics, fluctuation theorems and molecular machines. Rep. Prog. Phys. 75, 126001. 10.1088/0034-4885/75/12/126001 23168354

[B38] Sevick E. M. Prabhakar R. Williams S. R. Searles D. J. (2008). Fluctuation theorems. Annu. Rev. Phys. Chem. 59, 603–633. 10.1146/annurev.physchem.58.032806.104555 18393680

[B39] Sivak D. A. Crooks G. E. (2012). Thermodynamic metrics and optimal paths. Phys. Rev. Lett. 108, 190602. 10.1103/PhysRevLett.108.190602 23003019

[B40] Smith E. Morowitz H. J. (2004). Universality in intermediary metabolism. Proc. Natl. Acad. Sci. U.S.A. 101, 13168–13173. 10.1073/pnas.0404922101 15340153 PMC516543

[B41] Stanford N. J. Lubitz T. Smallbone K. Klipp E. Mendes P. Liebermeister W. (2013). Systematic construction of kinetic models from genome-scale metabolic networks. PLoS ONE 8:e79195. 10.1371/journal.pone.0079195 24324546 PMC3852239

[B42] Wittmann A. Suess B. (2012). Engineered riboswitches: expanding researchers’ toolbox with synthetic RNA regulators. FEBS Lett. 586, 2076–2083. 10.1016/j.febslet.2012.02.038 22710175

[B43] Xiao T. J. Hou Z. H. Xin H. W. (2009). Stochastic thermodynamics in mesoscopic chemical oscillation systems. J. Phys. Chem. B 113, 9316–9320. 10.1021/jp901610x 19518121

[B44] Zimmermann E. Seifert U. (2012). Efficiencies of a molecular motor: a generic hybrid model applied to the F-1-ATPase. New J. Phys. 14, 20 10.1088/1367-2630/14/10/103023

